# Survival of Six Probiotic Strains During Simulated Gastrointestinal Digestion in Fermented Sheep’s Milk Supplemented with Inulin and Sodium Butyrate

**DOI:** 10.3390/nu18142223

**Published:** 2026-07-08

**Authors:** Katarzyna Szajnar, Małgorzata Pawlos, Julita Drobniak, Agata Znamirowska-Piotrowska

**Affiliations:** Department of Dairy Technology, Faculty of Technology and Life Sciences, University of Rzeszów, Ćwiklińskiej 2D, 35-601 Rzeszów, Poland; mpawlos@ur.edu.pl (M.P.); jdrobniak@ur.edu.pl (J.D.); aznamirowska@ur.edu.pl (A.Z.-P.)

**Keywords:** in vitro digestion, probiotic bacteria, inulin, sodium butyrate

## Abstract

Background/Objectives: The aim of this study was to produce fermented sheep milk and to evaluate its microbiological properties. In addition, the survival of six probiotic strains was assessed using a standardized static in vitro digestion model. Methods: Fermented sheep milk samples supplemented with inulin or with the combination of inulin and sodium butyrate were analyzed using a standardized in vitro digestion procedure simulating oral, gastric, and intestinal phases. The survival rate of probiotic bacteria was additionally calculated. Results: The viable cell counts in sheep milk prior to digestion were significantly influenced by the bacterial strain used for fermentation. Analysis of variance demonstrated that probiotic survival during simulated gastrointestinal digestion was significantly affected by the investigated factors (bacterial strain, inulin supplementation, and supplementation with inulin and sodium butyrate), as well as by the interactions among these factors. The effects of supplementation on probiotic survival were strain-dependent. Conclusions: Considering the limited data available regarding the effects of sodium butyrate and inulin on probiotic survival in sheep milk matrices, the obtained results indicate that supplementation with these compounds may modulate the survival of probiotic strains during simulated gastrointestinal digestion in a strain-dependent manner. This study provides new insights into the influence of sodium butyrate and inulin on probiotic viability and gastrointestinal tolerance in fermented sheep milk.

## 1. Introduction

The ability of probiotic microorganisms to produce metabolites involved in the regulation of intestinal homeostasis is regarded as one of the major contributors to their health-promoting potential. Probiotics comprise numerous microbial strains, among which bacteria belonging to the genera *Bifidobacterium* and *Lactobacillus* are the most extensively investigated [1]. Representatives of the genus *Lactobacillus*, including *Lacticaseibacillus casei*, *Lactobacillus acidophilus*, and *Lacticaseibacillus rhamnosus*, are widely applied in dairy products and dietary supplements. These strains exhibit the ability to survive under acidic gastric conditions and to adhere to intestinal epithelial cells [2]. In turn, selected species of the genus *Bifidobacterium*, including *B. longum*, *B. bifidum*, and *B. breve*, beneficially modulate gut microbiota composition, support digestive processes, and contribute to the enhancement of host immune responses [3]. Since reduced numbers of *Bifidobacterium* spp. and butyrate-producing bacteria have been observed in the human colon of patients with various disorders, and because short-chain fatty acids produced by these microorganisms exert beneficial physiological effects, these bacteria are considered promising targets for the restoration and maintenance of intestinal homeostasis. Among the nutritional approaches used to promote the growth of bifidobacteria and butyrate-producing bacteria in the human colon, supplementation with probiotics and prebiotics remains the most extensively studied [4].

Bifidobacteria also support intestinal homeostasis through the production of acetate and lactate during carbohydrate fermentation. These metabolites play an important ecological role within the gut microbiota, as they can be further metabolized by butyrate-producing bacteria, leading to the formation of butyrate via cross-feeding interactions [5,6,7]. Studies employing genetic and metagenomic analyses have demonstrated that butyrate-producing microorganisms comprise a metabolically defined functional group rather than a single evolutionary lineage. In the colonic microbiota, the majority of bacteria capable of butyrate synthesis belong to the phylum Firmicutes, particularly to members of *Clostridium* clusters IV and XIVa, which are recognized as major contributors to intestinal butyrate production [8,9,10].

Butyrate plays a crucial role in maintaining intestinal health by influencing both host physiology and gut microbiota composition. Optimal concentrations of butyrate support colonocyte activity, strengthen intestinal barrier integrity, reduce inflammation, and promote microbial balance. Moreover, butyrate exhibits protective effects in intestinal disorders such as inflammatory bowel disease, graft-versus-host disease, and colorectal cancer, whereas reduced levels of butyrate or diminished populations of butyrate-producing bacteria are associated with impaired intestinal function and poorer health outcomes [11]. Butyrate is considered a particularly important short-chain fatty acid (SCFA) because it serves as the primary energy source for colonocytes. Additionally, it regulates histone acetylation by activating histone acetyltransferases (HATs) at low concentrations and inhibiting class I and II histone deacetylases (HDACs) at higher concentrations [12,13].

Among dairy matrices used for the development of functional foods, sheep milk is considered particularly valuable due to its high nutritional density and favorable technological properties. Compared with bovine milk, sheep milk contains higher concentrations of protein, fat, calcium, phosphorus, vitamins, and bioactive compounds, which may influence both the technological properties of fermented products and the survival of probiotic microorganisms during gastrointestinal transit [14,15]. Several authors have reported that the unique composition of sheep milk may enhance the resistance of probiotic microorganisms to gastrointestinal stress. The higher protein content and buffering capacity of sheep milk can reduce the detrimental effects of gastric acidity, whereas the fat fraction may contribute to enhanced bacterial tolerance to gastrointestinal stress conditions. Moreover, differences in protein structure and coagulum formation during digestion have been reported to affect digestion kinetics and microbial survival [15,16]. Fermented sheep milk has already been recognized as a suitable carrier for probiotic microorganisms. Kowalczyk et al. [17] reported that sheep milk supplemented with prebiotic ingredients supported the survival of probiotic bacteria during simulated gastrointestinal digestion. Similarly, several studies have highlighted the potential of sheep milk as a matrix for the development of functional probiotic and synbiotic products due to its favorable nutritional composition and technological properties [14,15].

Earlier investigations conducted by our research group also indicated that sheep milk supplemented with selected functional ingredients may effectively support probiotic survival during simulated digestion [14]. However, despite the growing interest in sheep milk-based functional products, limited information is available regarding the combined effect of inulin and sodium butyrate supplementation on the gastrointestinal survival of probiotic bacteria in fermented sheep milk.

Therefore, the aim of the present study was to evaluate the survival of six probiotic strains in fermented sheep milk supplemented with inulin and sodium butyrate using a static in vitro digestion model. The effects of inulin and sodium butyrate, applied individually and in combination, on the growth and survival of individual probiotic monocultures in fermented sheep milk were investigated.

A review of the available literature revealed a lack of studies addressing this specific research topic, highlighting the novelty of the present work. Considering the numerous reports demonstrating the beneficial effects of sodium butyrate on gastrointestinal function, we aimed to investigate whether its addition may influence the survival of probiotic bacteria during successive stages of simulated gastrointestinal digestion, including the oral, gastric, and intestinal phases. To achieve this, viable cell counts were determined in fermented sheep milk supplemented with sodium butyrate and compared with corresponding control samples without this additive.

The obtained results demonstrated that the applied supplements significantly affected the survival of the investigated probiotic strains. Moreover, the observed effects depended both on the type of supplementation and on the characteristics of the individual probiotic strain, confirming the strain-dependent nature of the response to the investigated factors.

## 2. Materials and Methods

### 2.1. Materials

Fermented milk was produced using commercial sheep milk (I Love My Sheep, Wartberg an der Krems, Austria). According to the manufacturer’s declaration, the composition per 100 mL was as follows: 6.0 g fat, 4.7 g lactose, 4.6 g protein, and 0.16 g salt. To evaluate the effect of supplementation, three formulations of fermented sheep milk were produced: a control sample, milk enriched with 4% (*w*/*w*) inulin, and milk supplemented with 4% (*w*/*w*) inulin combined with 0.06% (*w*/*w*) sodium butyrate. Inulin used in the study was a food-grade chicory-derived inulin obtained from TAR-GROCH-FIL (Filipowice, Poland). Sodium butyrate was supplied by OstroVit Sp. z o.o. (Zambrów, Poland) in the form of enteric microcapsules containing 30% butyric acid according to the manufacturer’s specification. The probiotic cultures *Bifidobacterium animalis* subsp. *lactis* BB-12, *Lacticaseibacillus casei* 431, *Lacticaseibacillus paracasei* L26, and *Lactobacillus acidophilus* LA-5 were obtained as freeze-dried direct-vat-set (DVS) cultures from Chr. Hansen (Hoersholm, Denmark). *Lactobacillus rhamnosus* Lr-32 was supplied by Danisco (DuPont, Copenhagen, Denmark) as a freeze-dried culture, whereas *Lactobacillus johnsonii* LJ Delvo^®^Pro was obtained from DSM (Delft, The Netherlands) as a frozen culture for direct inoculation.

Microbiological media and analytical reagents, including MRS agar and peptone water, were supplied by Biocorp (Warsaw, Poland) and Chempur (Piekary Śląskie, Poland). Reagents required for the simulated gastrointestinal digestion procedure, including α-amylase, mucin, pepsin, bile extract, and pancreatin, were purchased from Sigma-Aldrich (St. Louis, MO, USA). Additional analytical-grade reagents used for the in vitro digestion procedure, including Na_2_HPO_4_, K_2_HPO_4_, NaCl, HCl, and NaOH, were obtained from Chempur (Piekary Śląskie, Poland).

### 2.2. Sample Preparation

Three variants of fermented sheep milk were prepared: a control sample, milk supplemented with 4% inulin, and milk supplemented with 4% inulin and 0.06% sodium butyrate. The supplements were incorporated into the milk before technological processing according to the experimental design (Table 1).

All milk variants were homogenized at 60 °C under a pressure of 20 MPa and subsequently heat-treated at 85 °C for 10 min. After cooling to 37 ± 1 °C, the samples were inoculated with 5% (*w*/*w*) activated probiotic monocultures. The following strains were used: *Bifidobacterium animalis* subsp. *lactis* BB-12, *Lactobacillus johnsonii* LJ, *Lacticaseibacillus casei* 431, *Lacticaseibacillus paracasei* L26, *Lactobacillus acidophilus* LA-5, and *Lactobacillus rhamnosus* Lr32. Activated cultures were prepared in sterile milk at 37 ± 1 °C and used when the bacterial population reached approximately 9 log CFU g^−1^.

Following inoculation, the milk was distributed into sterile polypropylene containers (100 mL) and incubated at 37 °C until a final pH of 4.60 ± 0.10 was achieved. The fermented products were then cooled and stored at 5 °C in a refrigerated incubator (Cooled Incubator ILW 115, POL-EKO Aparatura, Wodzisław Śląski, Poland).

For each experimental variant, 500 mL of inoculated milk was prepared and divided into five 100 mL containers to provide sufficient material for microbiological analyses and simulated gastrointestinal digestion. All determinations were performed after 24 h of refrigerated storage.

### 2.3. In Vitro Digestion Protocol

A modified static in vitro gastrointestinal digestion model, based on a previously published protocol [17] for the assessment of probiotic survival, was used in the present study. The procedure consisted of consecutive oral, gastric, and intestinal phases performed under controlled laboratory conditions. For each fermented milk variant, 50 mL portions were subjected to the digestion procedure independently in triplicate.

The oral phase was initiated by combining 50 mL of fermented milk with 5 mL of simulated saliva. The salivary fluid contained Na_2_HPO_4_ (2.38 g/L), K_2_HPO_4_ (0.19 g/L), NaCl (8.0 g/L), mucin (M2378, from porcine stomach, type II, 100 mg/L), and α-amylase (A3306, heat stable, 200 U/L, 200 g/L). The pH of the mixture was adjusted to 6.75 ± 0.20, and samples were incubated at 37 °C for 2 min with continuous shaking (90 rpm).

To simulate gastric digestion, pepsin (P7000, from porcine gastric mucosa, 535 U/L, 13.08 mg/L) was added to each sample, and the pH was reduced to 2.00 ± 0.20 using 12 M HCl. Samples were then incubated for 2 h at 37 °C under constant agitation (90 rpm).

The intestinal phase was performed by adding 5 mL of pancreatin solution (P7545, from porcine pancreas, 8 × USP specifications, 100 U/mL, 4 g/L) and 5 mL of porcine bile extract (B8631, 25 g/L) to the gastric digesta. Subsequently, the pH was adjusted to 7.00 ± 0.20 with 1 M NaOH, and incubation was continued for a further 2 h at 37 °C with shaking at 90 rpm. Samples were collected immediately after completion of each digestion stage and used for microbiological analyses described in Section 2.4.

### 2.4. Microbiological Analysis

The viability of probiotic bacteria was determined before digestion and after each stage of the simulated gastrointestinal digestion procedure (oral, gastric, and intestinal phases).

For microbiological enumeration, samples were serially diluted in sterile 0.1% peptone water and plated on MRS agar with Tween 80. Depending on the expected bacterial population, appropriate dilution levels were selected for analysis. The inoculated plates were incubated anaerobically at 37 °C for 72 h using the GENbox anaerobic system (Biomerieux, Warsaw, Poland).

After incubation, bacterial colonies were counted using a TYPE J-3 colony counter (Chemland, Stargard Szczeciński, Poland), and the results were expressed as log CFU g^−1^.

The survival rate of probiotic bacteria was calculated according to the following equation [17]:

$$\mathrm{Survival}\;\mathrm{rate}\;(\%)\;=\;\frac{Viable\;counts\;in\;digested\;sample}{Viable\;counts\;in\;undigested\;sample}\times 100$$

### 2.5. Statistical Analysis

Statistical analyses were performed using Statistica 13.1 software (StatSoft, Tulsa, OK, USA). The obtained results were expressed as mean values ± standard deviation (SD). Differences between means were evaluated using Tukey’s post hoc test at a significance level of *p* ≤ 0.05. Additionally, one-way and multifactor analyses of variance (ANOVA) were conducted to determine the effects of the investigated factors and their interactions. Three independent production batches were prepared for each experimental variant. For each production batch, microbiological analyses were carried out in duplicate at each stage of the simulated gastrointestinal digestion procedure.

## 3. Results

The results of probiotic viability determined before simulated digestion and after the successive stages of in vitro digestion (oral cavity, gastric phase, and intestinal phase) are presented in Table 2.

### 3.1. Before Digestion

**Figure 1:**
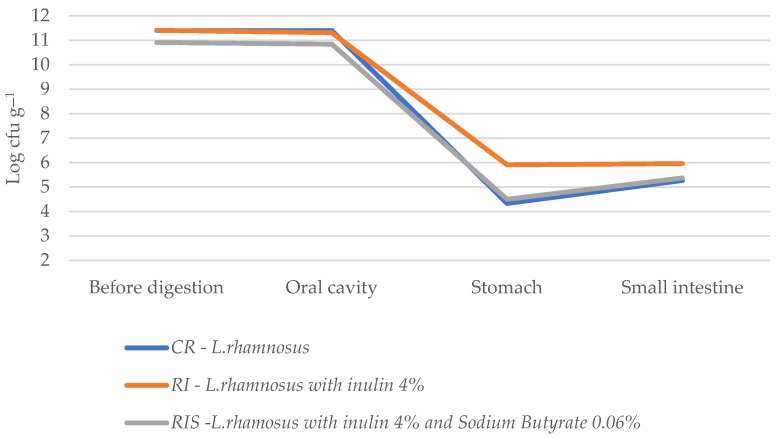
Changes in the viable counts of *L. rhamnosus* before and during the different stages of simulated digestion.

**Figure 2:**
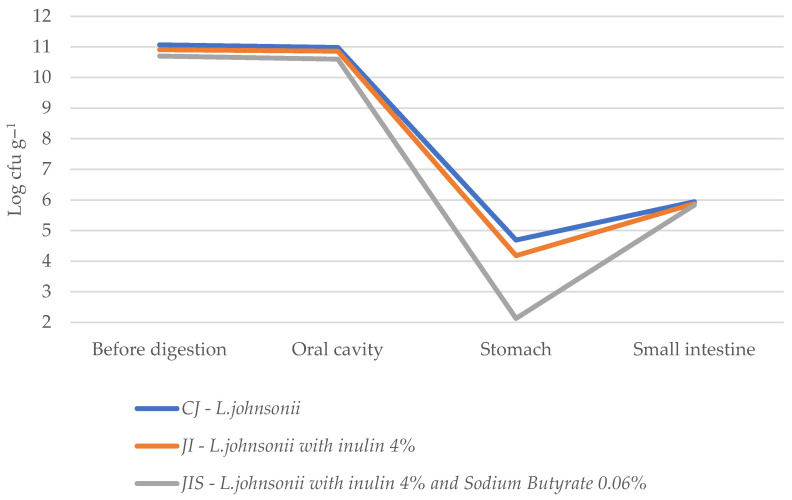
Changes in the viable counts of *L. johnsonii* before and during the different stages of simulated digestion.

**Figure 3:**
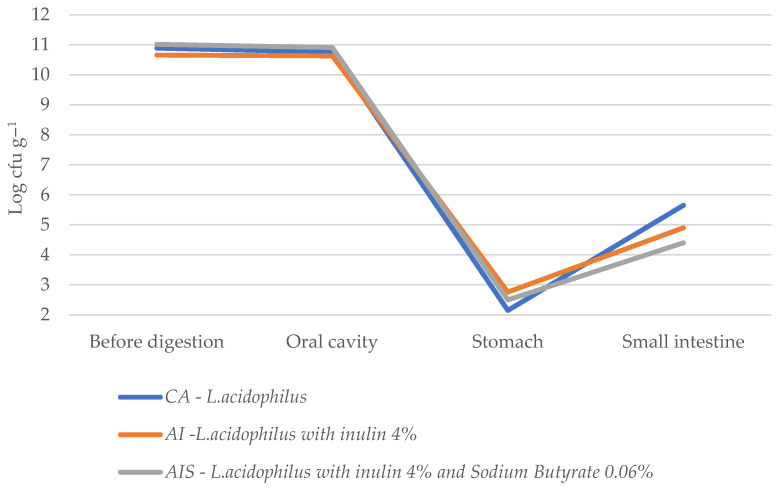
Changes in the viable counts of *L. acidophilus* before and during the different stages of simulated digestion.

**Figure 4:**
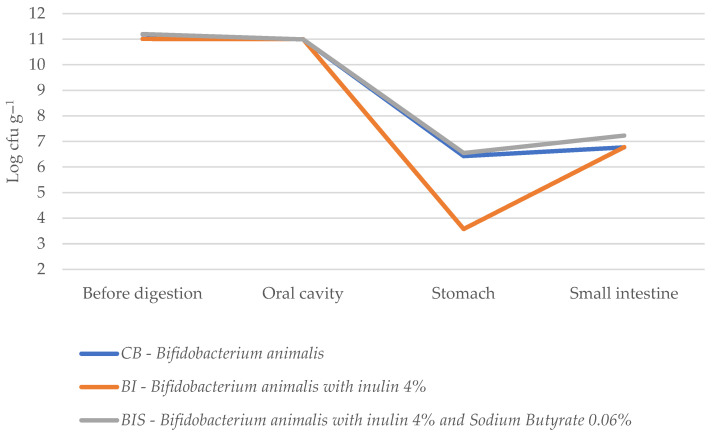
Changes in the viable counts of *B. animalis* before and during the different stages of simulated digestion.

**Figure 5:**
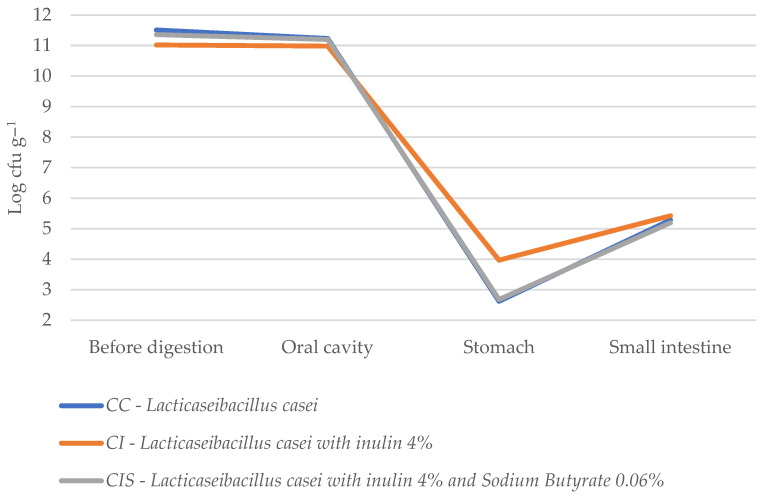
Changes in the viable counts of *L. casei* before and during the different stages of simulated digestion.

**Figure 6:**
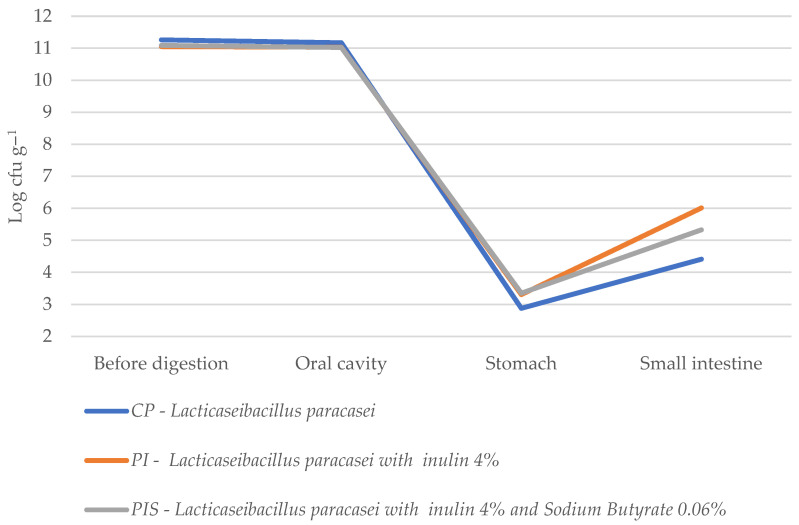
Changes in the viable counts of *L. paracasei* before and during the different stages of simulated digestion.

Changes in the viable counts of probiotic bacteria before and during the different stages of simulated digestion are presented in Figure 1, Figure 2, Figure 3, Figure 4, Figure 5 and Figure 6.

The highest viable counts prior to digestion were observed in control sheep milk samples CC fermented by *L. casei* and CR fermented by *L. rhamnosus*. Significantly lower (*p* ≤ 0.05) bacterial counts were recorded in sheep milk fermented by *L. johnsonii*, *L. acidophilus*, *L. paracasei*, and *B. animalis* subsp. *Lactis*. These observations were confirmed by ANOVA analysis (Table 3), which demonstrated that the probiotic strain used for fermentation significantly affected bacterial counts in sheep milk prior to digestion (*p* = 0.0000).

An important aspect of the present study was the evaluation of whether supplementation applied before fermentation influenced probiotic populations in fermented milk prior to digestion. It was demonstrated that only in sheep milk RIS fermented by *L. rhamnosus* did supplementation with inulin and sodium butyrate significantly reduce probiotic counts. In the remaining experimental variants, no such effect was observed compared with the corresponding control samples.

### 3.2. Oral Cavity

As expected, during the oral phase, probiotic counts in all analyzed samples did not differ significantly compared with undigested fermented milk samples. The negative effect of inulin and sodium butyrate supplementation on probiotic counts in RIS milk was still observed at this stage (Figure 1, Figure 2, Figure 3, Figure 4, Figure 5 and Figure 6).

### 3.3. Stomach

Gastric digestion under acidic conditions typically lasts from 1 to 3 h. In the present study, after 2 h of exposure to simulated gastric juice, a significant reduction in probiotic populations was observed in all analyzed milk samples. *L. acidophilus* proved to be the least resistant strain to gastric conditions, with population reductions ranging from 7.90 log cfu g^−1^ in AI samples to 8.74 log cfu g^−1^ in CA samples (Figure 3). Similarly, *L. paracasei* and *L. casei* also demonstrated relatively low resistance to gastric juice, exhibiting reductions of 7.74–8.38 log cfu g^−1^ and 7.05–8.89 log cfu g^−1^, respectively (Figure 6).

It should be emphasized that inulin supplementation positively affected probiotic counts during the gastric phase only in samples fermented by *L. rhamnosus* (RI), *L. casei* (CI), and *L. paracasei* (PI) (Figure 1, Figure 5 and Figure 6). Moreover, supplementation with inulin and sodium butyrate significantly increased the survival of *L. paracasei* during the gastric phase compared with the control CP sample (Figure 6). In contrast, significantly lower probiotic counts after gastric exposure were recorded in JIS milk fermented by *L. johnsonii* supplemented with inulin and sodium butyrate compared with JI and CJ samples (Figure 2). Supplementation did not significantly affect *L. acidophilus* counts during the gastric phase (Figure 3).

### 3.4. Small Intestine

During the intestinal phase, a significant increase in probiotic counts compared with the gastric phase was observed in most experimental variants. The only exception was the RI sample fermented by *L. rhamnosus*, in which probiotic counts remained at approximately 5.9 log cfu g^−1^ in both digestion stages (Figure 1). A negative effect of supplementation with inulin and with inulin combined with sodium butyrate on bacterial counts during the intestinal phase was observed for samples fermented by *L. acidophilus* (AI and AIS).

A beneficial effect of supplementation with inulin and sodium butyrate was demonstrated in BIS milk fermented by *B. animalis* subsp. *lactis* compared with the corresponding control sample. This variant also exhibited the highest probiotic counts detected during the intestinal phase in the entire experiment, exceeding 5 log cfu g^−1^ (Figure 4). Significant beneficial effects of supplementation on probiotic counts were also observed for PI and PIS samples (Figure 5).

### 3.5. Survival Rates (%)

**Figure 7:**
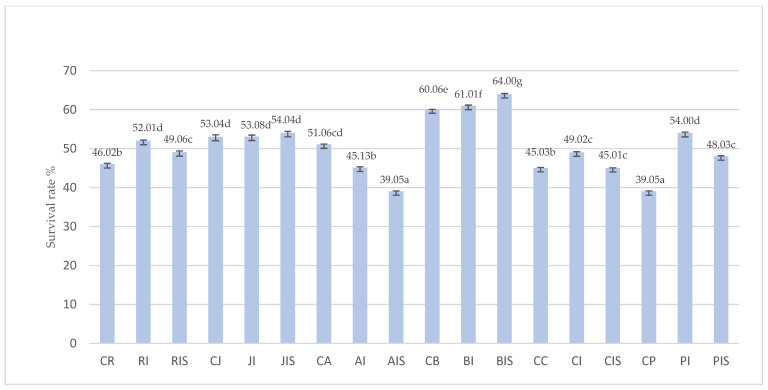
Survival rates (%) in milk fermented by probiotic bacteria. Mean ± standard deviation; ^a–g^—mean values denoted by different letters indicate statistically significant differences at *p* ≤ 0.05. CR—*L. rhamnosus*; RI—*L. rhamnosus* with 4% inulin; RIS—*L. rhamnosus* with 4% inulin and 0.06% sodium butyrate; CJ—*L. johnsonii*; JI—*L. johnsonii* with 4% inulin; JIS—*L. johnsonii* with 4% inulin and 0.06% sodium butyrate; CA—*L. acidophilus*; AI—*L. acidophilus* with 4% inulin; AIS—*L. acidophilus* with 4% inulin and 0.06% sodium butyrate; CB—*B. animalis*; BI—*B. animalis* with 4% inulin; BIS—*B. animalis* with 4% inulin and 0.06% sodium butyrate; CC—*L. casei*; CI—*L. casei* with 4% inulin; CIS—*L. casei* with 4% inulin and 0.06% sodium butyrate; CP—*L. paracasei*; PI—*L. paracasei* with 4% inulin; PIS—*L. paracasei* with 4% inulin and 0.06% sodium butyrate.

From both technological and consumer perspectives, probiotic survival throughout gastrointestinal digestion relative to initial counts before digestion represents an important parameter. The lowest probiotic survival was observed in control sheep milk CP fermented by *L. paracasei* and in milk supplemented with inulin and sodium butyrate fermented by *L. acidophilus*, where survival rates reached only 39% (Figure 7).

In milk fermented by *L. acidophilus*, supplementation exerted a negative effect on probiotic survival, as inulin reduced survival by 5.93%, while supplementation with inulin and sodium butyrate decreased survival by as much as 12.01% compared with the control sample. In contrast, supplementation significantly improved the survival of *L. paracasei*, with increases of 14.95% for inulin supplementation and 8.98% for inulin combined with sodium butyrate. Increased survival was also observed for *L. rhamnosus* (RI: +5.99%; RIS: +3.04%) and *B. animalis* subsp. *lactis* (BI: +0.95%; BIS: +3.94%) compared with the corresponding control samples.

Analysis of variance (Table 3) demonstrated that probiotic survival was significantly affected by the investigated factors, including probiotic strain, inulin supplementation, supplementation with inulin and sodium butyrate, and the interactions among these three factors.

## 4. Discussion

Among the short-chain fatty acids produced in the human colon, butyrate is considered one of the most physiologically important metabolites due to its broad spectrum of biological activities. This compound serves as the principal energy substrate for colonocytes, contributes to the maintenance of epithelial barrier integrity, and plays a key role in the regulation of immune and inflammatory responses. In recent years, increasing attention has been directed toward nutritional strategies capable of enhancing intestinal butyrate production because disturbances in butyrate metabolism have been associated with various gastrointestinal and systemic disorders. The bifidogenic properties of inulin-type fructans (ITFs) and arabinoxylan oligosaccharides (AXOS) are thought to arise from cooperative and strain-specific mechanisms of carbohydrate utilization within intestinal microbial communities. Through the fermentation of these substrates, bifidobacteria produce metabolites that can subsequently be utilized by butyrate-producing microorganisms via metabolic cross-feeding interactions. Consequently, supplementation with selected prebiotics may contribute not only to an increase in bifidobacterial populations but also to enhanced butyrate synthesis within the colon. Species belonging to the genus Bifidobacterium therefore play a particularly important role in maintaining microbial homeostasis and supporting intestinal health [18,19,20]. Reduced abundance of these species in the large intestine has been associated with numerous disorders. Furthermore, bifidobacteria have been shown to interact with other members of the gut microbiota, including butyrate-producing bacteria, through cross-feeding interactions. Moreover, decreased butyrate concentrations and reduced abundance of butyrate-producing bacteria in the human colon have also been linked to various disorders.

The maintenance of adequate probiotic populations throughout product storage is considered essential to ensure the delivery of health-promoting effects to consumers. In this context, Ozturkoglu-Budak et al. [21] demonstrated that the populations of *L. acidophilus* and *B. animalis* in fermented milk remained above 8 and 7 log cfu/mL, respectively, during the entire storage period. The bacterial counts obtained in the present study are in agreement with these observations and support the generally accepted recommendation that probiotic dairy products should contain at least 7 log cfu/mL of viable microorganisms to exert beneficial physiological effects [22]. In the same study, supplementation with inulin differing in degree of polymerization stimulated the growth of both probiotic strains. Similar findings have been reported by Biedrzycka and Bielecka [23] as well as Rossi et al. [24], who demonstrated that bifidobacteria efficiently utilize short-chain fructans. The ability of bifidobacteria to metabolize monosaccharides released during inulin degradation through the fructose-6-phosphate shunt pathway has also been documented [25]. Nevertheless, the influence of inulin appears to be strain-dependent, as Ozer et al. [26] did not observe growth stimulation of *L. acidophilus* in acidophilus–bifidus yogurt, which was subsequently confirmed by Bedani et al. [27].

Kocer et al. [28] demonstrated that *L. acidophilus* populations decreased by approximately 2–3 logarithmic cycles after 2 h of gastric digestion. According to the authors, this finding indicates high sensitivity of *L. acidophilus* to simulated gastric juice containing HCl and pepsin, as the greatest reduction in probiotic survival was consistently observed during the gastric stage. This phenomenon may be associated with strain-dependent tolerance of lactic acid bacteria to acidic conditions, which additionally depends on external factors, culture medium composition, and incubation conditions [29]. Various approaches have been proposed to improve probiotic survival both during gastrointestinal transit and throughout product storage until consumption, including the use of mixed probiotic cultures, microencapsulation, prebiotic supplementation, and alternative food matrices [30,31,32,33,34]. Among these strategies, particular attention has been paid to prebiotics, which are widely regarded as an effective method for improving probiotic viability and resistance to gastrointestinal conditions.

Our previous investigation examining fermented milk supplemented with inulin and sodium butyrate also demonstrated that the response of probiotic bacteria to these functional additives was highly strain-dependent [35]. Among the strains evaluated in that study, *L. acidophilus*, *B. animalis* subsp. *lactis*, and *L. johnsonii* exhibited the greatest resistance to simulated gastrointestinal conditions, maintaining survival rates exceeding 55% of the initial bacterial population. Supplementation with inulin exerted the most pronounced beneficial effect on the survival of *L. paracasei*, *B. animalis* subsp. *lactis*, and *L. acidophilus*, whereas the combination of inulin and sodium butyrate proved particularly advantageous for *L. casei* and *L. acidophilus*. Interestingly, the survival of *L. johnsonii* remained relatively unaffected by the supplementation strategy, suggesting that the response to prebiotic and postbiotic compounds may depend on specific physiological and metabolic characteristics of individual probiotic strains. Taken together, the findings of the previous study [35] and the results obtained in the present work indicate that the effectiveness of inulin and sodium butyrate cannot be generalized across probiotic species and should be evaluated individually for each strain under investigation.

### Limitations

It should be emphasized that the static in vitro digestion model employed in the present study, despite its widespread use and high degree of standardization, represents a limitation of the research. This model is unable to fully reproduce the complex and dynamic processes occurring within the human gastrointestinal tract. In vivo conditions are influenced by numerous physiological factors, including individual variations in the composition and activity of digestive enzymes; fluctuations in pH throughout different sections of the gastrointestinal tract; and variations in gastric emptying rate, intestinal peristalsis, and mucus secretion, as well as interactions among food components, the intestinal microbiota, and host cells.

In contrast to in vivo conditions, static in vitro digestion models are based on strictly defined and constant experimental parameters, which do not allow for the full simulation of the dynamic changes occurring during the digestive process. Furthermore, these models do not account for the influence of the host immune response, nutrient absorption across the intestinal epithelium, or the complex interactions between the investigated probiotic strains and the indigenous gut microbiota.

Despite these limitations, standardized in vitro digestion models are recognized as valuable research tools that enable the preliminary assessment of probiotic microorganism survival and the comparison of the effects of different technological factors and food ingredients on their stability during simulated gastrointestinal transit. The results obtained provide important screening information; however, their full validation requires further investigations employing dynamic digestion models as well as in vivo studies [36,37,38].

## 5. Conclusions

The results obtained in the present study demonstrated that the survival of probiotic strains during simulated gastrointestinal digestion was strongly strain-dependent and significantly influenced by the applied supplementation strategy. Probiotic viability prior to digestion varied depending on the bacterial strain used for fermentation, whereas the oral digestion stage had no significant effect on bacterial counts. In contrast, exposure to simulated gastric conditions resulted in a substantial reduction in probiotic populations in all analyzed samples, confirming the gastric phase as the most critical stage affecting bacterial survival.

Among the investigated strains, *Lactobacillus acidophilus* exhibited the lowest resistance to gastrointestinal stress conditions, while *Bifidobacterium animalis* subsp. *lactis* showed the highest survival during intestinal digestion following supplementation with inulin and sodium butyrate. The effects of supplementation were strain-dependent, as inulin and sodium butyrate improved survival in selected strains but negatively affected others. Analysis of variance further confirmed that probiotic survival was significantly influenced by bacterial strain, supplementation type, and interactions among these factors.

Overall, these findings provide new insights into the effects of inulin and sodium butyrate on probiotic viability and gastrointestinal tolerance and highlight the importance of strain-specific approaches when designing probiotic formulations intended to improve bacterial persistence under gastrointestinal stress conditions.

## Figures and Tables

**Table 1:** Milk groups obtained in the experiment.

Bacterial Strain	Control Group	Group with 4% Inulin	Group with 4% Inulin and 0.06% Sodium Butyrate
*Lacticaseibacillus paracasei* L26 (*L. paracasei*)	CP	PI	PIS
*Lacticaseibacillus casei* 431 (*L. casei*)	CC	CI	CIS
*Bifidobacterium animalis* ssp. *lactis* BB-12 (*B. animalis*)	CB	BI	BIS
*Lactobacillus johnsonii* LJ (*L. johnsonii*)	CJ	JI	JIS
*Lactobacillus acidophilus* LA-5 (*L. acidophilus*)	CA	AI	AIS
*Lactobacillus rhamnosus* Lr32 (*L. rhamnosus*)	CR	RI	RIS

**Table 2:** Viable counts of bacteria (log cfu g^−1^) in milk fermented by probiotic bacteria before digestion, in the oral cavity, stomach, and small intestine.

Probiotic Strain	Experimental Batch	Experimental Stage			
		Before Digestion	Oral Cavity	Stomach	Small Intestine
*L. rhamnosus* Lr32	CR—control	11.40 ^cA^ ± 0.02	11.40 ^dA^ ± 0.21	4.33 ^cC^ ± 0.28	5.27 ^bB^ ± 0.36
	RI—4% inulin	11.41 ^cA^ ± 0.31	11.31 ^cdA^ ± 0.52	5.91 ^dB^ ± 0.10	5.96 ^bcB^ ± 0.13
	RIS—4% inulin and 0.06% sodium butyrate	10.91 ^aA^ ± 0.47	10.84 ^bA^ ± 0.08	4.50 ^cC^ ± 0.10	5.37 ^bB^ ± 0.22
*L. johnsonii* LJ	CJ—control	11.07 ^abA^ ± 0.17	10.98 ^bA^ ± 0.13	4.69 ^cC^ ± 0.39	5.94 ^bcdB^ ± 0.97
	JI—4% inulin	10.91 ^aA^ ± 0.06	10.86 ^bA^ ± 0.16	4.18 ^cB^ ± 0.13	5.88 ^bB^ ± 0.86
	JIS—4% inulin and 0.06% sodium butyrate	10.70 ^aA^ ± 0.46	10.60 ^abA^ ± 0.14	2.13 ^aC^ ± 0.99	5.83 ^bB^ ± 0.74
*L. acidophilus* LA-5	CA—control	10.89 ^aA^ ± 0.16	10.76 ^bA^ ± 0.02	2.15 ^aC^ ± 0.67	5.65 ^bB^ ± 0.16
	AI—4% inulin	10.66 ^aA^ ± 0.40	10.63 ^aA^ ± 0.09	2.76 ^aC^ ± 0.11	4.90 ^aB^ ± 0.15
	AIS—4% inulin and 0.06% sodium butyrate	11.02 ^aA^ ± 0.15	10.91 ^bcA^ ± 0.07	2.50 ^aC^ ± 0.14	4.40 ^aB^ ± 0.63
*B. animalis* ssp. *lactis* BB-12	CB—control	11.19 ^bA^ ± 0.11	10.99 ^bcA^ ± 0.40	6.43 ^eC^ ± 0.06	6.77 ^dB^ ± 0.09
	BI—4% inulin	11.01 ^abA^ ± 0.21	11.00 ^cA^ ± 0.10	3.58 ^bC^ ± 0.28	6.78 ^dB^ ± 0.05
	BIS—4% inulin and 0.06% sodium butyrate	11.20 ^bA^ ± 0.10	10.99 ^bcA^ ± 0.18	6.55 ^eC^ ± 0.05	7.23 ^eB^ ± 0.20
*L. casei* 431	CC—control	11.51 ^cA^ ± 0.14	11.23 ^cA^ ± 0.19	2.62 ^ac^ ± 0.42	5.28 ^bcB^ ± 0.90
	CI—4% inulin	11.02 ^abA^ ± 0.17	10.98 ^bcA^ ± 0.11	3.97 ^bC^ ± 0.20	5.42 ^bB^ ± 0.52
	CIS—4% inulin and 0.06% sodium butyrate	11.36 ^cA^ ± 0.09	11.20 ^cA^ ± 0.12	2.68 ^aC^ ± 0.29	5.19 ^bB^ ± 0.21
*L. paracasei* L26	CP—control	11.26 ^bA^ ± 0.09	11.17 ^cA^ ± 0.15	2.88 ^aC^ ± 0.18	4.41 ^aB^ ± 1.02
	PI—4% inulin	11.05 ^aA^ ± 0.14	11.03 ^cA^ ± 0.15	3.31 ^bC^ ± 0.42	6.01 ^cB^ ± 0.51
	PIS—4% inulin and 0.06% sodium butyrate	11.09 ^abA^ ± 0.11	11.02 ^cA^ ± 0.09	3.35 ^bC^ ± 0.92	5.33 ^bB^ ± 0.11

Mean ± standard deviation; ^a–e^—mean values denoted in columns by different letters differ statistically significantly at *p* ≤ 0.05; ^A–C^—mean values in rows denoted by different letters differ significantly at *p* ≤ 0.05.

**Table 3:** Analysis of variance (ANOVA) *p*-value on the effect of probiotic strains, inulin, and inulin and sodium butyrate on viable counts of bacteria in milk fermented by probiotic bacteria before digestion in the oral cavity, stomach, and small intestine.

		Probiotic Strains *p*-Values	Inulin *p*-Values	Inulin and Sodium Butyrate *p*-Values	Probiotic Strains * Inulin *p*-Values	Probiotic Strains * Inulin and Sodium Butyrate *p*-Values	Inulin * Inulin and Sodium Butyrate *p*-Values	Probiotic Strains * Inulin * Inulin and Sodium Butyrate *p*-Values
Viable counts of bacteria in fermented by probiotic bacteria	Before digestion	0.0000 ↑	0.7396	0.0002 ↑	0.0511	0.0024 ↑	0.0520	0.0544
	Oral cavity	0.0000 ↑	0.0005 ↑	0.1704	0.1923	0.8242	0.1299	0.1289
	Stomach	0.0000 ↑	0.4211	0.4609	0.0002 ↑	0.0009 ↑	0.2789	0.7171
	Small intestine	0.0000 ↑	0.0529	0.0551	0.0003 ↑	0.0554	0.5533	0.0621
Survival rate		0.0001 ↑	0.0412 ↑	0.0312 ↑	0.0012 ↑	0.0491 ↑	0.0510	0.0421 ↑

Probiotic Strains * Inulin = interaction ↑; Probiotic Strains * Inulin and Sodium butyrate = interaction ↑; Inulin * Inulin and Sodium butyrate = interaction ↑; Probiotic Strains * Inulin * inulin and Sodium butyrate = interaction ↑; *p* < 0,05 indicates significant effect.

## Data Availability

The original contributions presented in this study are included in the article. Further inquiries can be directed to the corresponding author.
